# Incidence, Clinical Characteristics, and Outcomes of Clostridium difficile Infection in a Tertiary Care Center in Bahrain

**DOI:** 10.7759/cureus.57381

**Published:** 2024-04-01

**Authors:** Safa Alkhawaja, Tamer T Abo Arisheh, Rommel Acunin, Fadheela A Alawi, Abdulrahman Sharaf, Mahmood Alawainati, Alaa M Alzamrooni, Husain A Husain, Sumaya Alsalah

**Affiliations:** 1 Internal Medicine, Salmaniya Medical Complex, Manama, BHR; 2 Infection Prevention and Control, Government Hospitals Bahrain, Manama, BHR; 3 Infectious Diseases, Salmaniya Medical Complex, Manama, BHR; 4 Pharmacy, Salmaniya Medical Complex, Manama, BHR

**Keywords:** clostridium difficile, clostridioides, atlas score, antimicrobial therapy, bahrain, cdi

## Abstract

Background

*Clostridioides difficile* infection (CDI) represents a significant healthcare challenge associated with antibiotic use and healthcare settings. While healthcare facility-onset CDI (HO-CDI) rates have been extensively studied, the incidence and risk factors of CDI in various settings, including the community, require further investigation.

Aim

This study aims to examine the incidence rates of CDI in a major governmental hospital in Bahrain, identify risk factors for CDI, and assess the effectiveness of infection control measures.

Method

We conducted a retrospective study at the Salmaniya Medical Complex (SMC), analyzing all confirmed cases of CDI over a 30-month period from January 2021 to June 2023. CDI cases were screened using glutamine dehydrogenase antigen detection and confirmed using molecular assays like polymerase chain reaction and/or toxin assays for confirmation. The study categorized CDI cases based on their onset (hospital or community) and explored associated risk factors, including antibiotic use, proton pump inhibitor (PPI) therapy, and patient demographics. Infection control practices were also evaluated for their role in managing CDI.

Results

About 57 new CDI cases were identified during the study period, with a HO-CDI incidence rate of 0.5 per 10,000 patient days. While HO-CDI rates remained stable, community-onset (CO)-CDI cases increased. The median patient age was 61.8 years, without notable differences between genders. Key risk factors for CDI were antimicrobial therapy, use of acid-reducing agents, age, and underlying comorbidities. The mortality rate stood at 35.1%. The ATLAS score (i.e., age, treatment with antibiotics, leukocyte count, albumin level, and serum creatinine) was a reliable predictor of mortality. Critical care admission and low albumin levels emerged as significant independent risk factors for mortality.

Conclusions

The study demonstrates a low incidence rate of HO-CDI at SMC, attributed to effective infection control and antibiotic stewardship programs. The overall CDI rate increased during the study period, driven by a rise in CO cases; further investigating the risk factors among this category in our study revealed that most patients were exposed to antibiotic therapy within the past three months of their CDI diagnosis. The rise in CO-CDI cases underscores the need for broader community-based interventions and awareness regarding antibiotic and PPI use.

## Introduction

*Clostridioides difficile*, formerly known as *Clostridium difficile*, is a bacterium that colonizes the intestinal tract. It can lead to antibiotic-associated diarrhea when antibiotic treatment disrupts the normal gut flora. *C. difficile* infection (CDI) is often associated with healthcare settings and has caused numerous outbreaks in hospitals due to its resilience in hospital environments and resistance to common cleaning agents and alcohol-based hand sanitizers. Its mode of transmission is fecal-oral, and human infection in healthcare institutions is usually through contaminated environmental surfaces and through the hands of healthcare professionals. Therefore, isolation of infected cases, in addition to appropriate surface cleaning and proper hand hygiene practice by healthcare professionals, is crucial to decrease the risk of CDI among hospitalized patients [1].

While initially considered solely a healthcare-associated infection, the epidemiology of CDI has shifted over the past decade, with one-third of cases arising outside of healthcare facilities [2-4]. US population-based surveillance data for CDI in 2019 revealed a crude overall incidence rate of CDI of 121.2 cases per 100,000 persons, with a higher incidence of community-associated cases (63.2 cases per 100,000 persons) compared with healthcare-associated cases (57.9 cases per 100,000 persons) [5].

The incidence of CDI in hospitals varies globally. A 2020 meta-analysis by Marra et al. [6] reported an incidence of 8.3 cases per 10,000 patient days in the United States, a finding echoed by studies from other Western countries indicating a rising trend over the last 10 years [7]. In contrast, data from the Middle East, especially in the Arabian Gulf countries, remain scarce.

The most commonly encountered risk factors for CDI are old age, antibiotics exposure, use of acid-suppressing agents, and presence of comorbidities [8]. Identifying these risk factors among hospitalized patients is important to predict the risk of acquisition; hence, working on minimizing the exposure to other modifiable factors, such as antibiotics and acid suppression therapy, with re-emphasis on the importance of periodic review of such therapies among inpatients for its necessity of continuation.

Certain antibiotic usage has been linked more frequently to CDI, such as penicillins, cephalosporins, carbapenems, and fluoroquinolones [9,10]. Mortality rates for CDI range from 2% to 30%, with higher rates among patients with comorbidities or in critical care units [11].

Several tools were proposed, including the age, treatment with antibiotics, leukocyte count, albumin level, and serum creatinine (ATLAS) score, to predict outcomes of CDI by evaluating said factors [12]. Treatment for CDI typically involves metronidazole, vancomycin, or fidaxomicin, though studies have indicated a higher failure rate with metronidazole monotherapy [3].

This study is the first to assess CDI incidence, risk factors, and outcomes in the Kingdom of Bahrain. It aims to determine the rate of CDI among inpatients at Salmaniya Medical Complex (SMC), identify risk factors, and evaluate treatment outcomes, contributing valuable insights into CDI risk factors to inform prevention strategies.

## Materials and methods

Study population and setting

This study was conducted at SMC, Bahrain’s main governmental hospital, with a capacity of 1,200 beds. The study included all inpatients with confirmed CDI through positive stool tests from January 2021 to June 2023. Our microbiology laboratory performed *C. difficile* stool analysis upon the physician’s request, accepting only unformed stool specimens. Initial screening involved Glutamine Dehydrogenase antigen detection and molecular assays (polymerase chain reaction (PCR) and/or toxin assays) for confirmation. Data were sourced from the SMC Infection Control Unit, which records all positive CDI cases. Researchers then collected further patient information from the electronic medical record in the National Health Information Program (I-SEHA) and compiled it in a datasheet.

Study design

This observational retrospective study included all inpatients with confirmed CDI. We defined incident CDI based on the specimen date relative to the patient’s last positive test, classifying any positive test obtained more than eight weeks after a previous positive test (or with no prior positive test documented) as an incident case. Recurrent CDI was defined as any positive test obtained two to eight weeks after the most recent positive test [3].

CDI cases were categorized based on the admission date and acquisition as follows: community-onset (CO), positive tests in inpatients within three days of admission; community-onset healthcare facility-associated (CO-HCFA), positive tests in inpatients within three days of admission and discharge from the facility within four weeks prior; healthcare facility-onset (HO), positive tests collected more than three days after admission [3]. The primary outcome was mortality, determined by the CDI patient’s status at discharge (alive/dead). Other outcomes included CDI complications during the hospital stay and recurrence within six months after initial diagnosis.

Statistical analysis

Descriptive statistics were used to report response proportions, numbers, and percentages for categorical variables as well as means and standard deviations for continuous variables. We analyzed the relationship between outcomes, patient demographics, and clinical characteristics using Fisher’s Exact test and an independent sample t-test. Significant findings led to multivariate regression analysis to identify independent risk factors for mortality. Statistical significance was set at p<0.05 and analyses were conducted using IBM SPSS Statistics for Windows, Version 26 (Released 2019; IBM Corp., Armonk, New York, United States).

Ethical approval

The Research Ethics Committee of Government Hospitals of Bahrain approved this study on July 18, 2023 (Approval No. 83180723). As a retrospective, non-interventional study involving no patient data disclosure, neither ethical considerations nor patient consent was required.

## Results

Incidence rate and categorization of CDI

**Figure 1:**
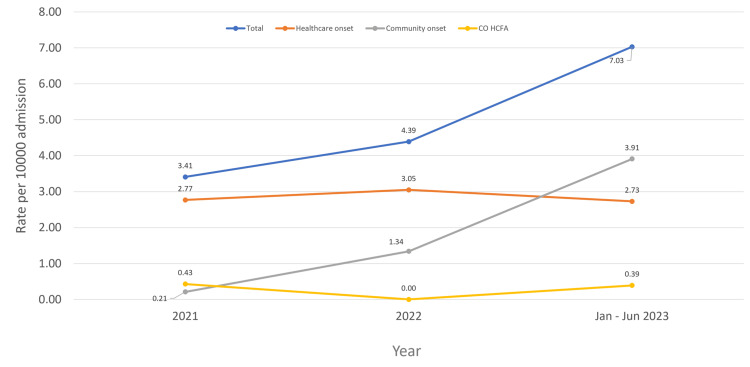
Rate of CDI per 10000 in SMC, Bahrain SMC: Salmaniya Medical Complex; CDI: *Clostridioides difficile* infection

During the study, 57 patients were identified with incident CDI. About 36 patients (63.2%) were categorized as HO, having positive tests for CDI more than three days after admission to SMC. Eighteen patients (31.6%) were CO with positive tests within the first three days of admission. Three cases (5.2%) were CO-HCFA, with positive tests within three days of admission and a discharge from SMC less than four weeks prior to the stool specimen collection. The incidence rate of HO-CDI at SMC, calculated over 711,466 inpatient days, was 0.5 per 10,000 patient days (Table 1). Figure 1 illustrates that the overall CDI rate increased during the study period, with CO cases rising in parallel. In contrast, HO and CO-HCFA cases remained relatively consistent.

**Table 1:** Frequencies and rates of CDI in SMC, Bahrain *Inpatient facility CDI healthcare facility-onset incident rate: Number of all incident HO-CDI lab ID events per year in facility x 10000/number of patient days for the facility in the same period **Inpatient facility CDI combined incident rate: Number of all incident HO and CO-HCFA CDI lab ID events per year in facility x 10000/number of patient days for the facility in the same period CDI: *Clostridioides difficile* infection; SMC: Salmaniya Medical Complex; CO: Community-onset; HO: healthcare facility-onset; HCFA: Healthcare facility-associated

Type of CDI and rate	2021 (Jan-Dec)	2022 (Jan-Dec)	2023 (Jan-June)	Total/average
CO	1	7	10	18
CO-HCFA	2	0	1	3
HO	13	16	7	36
Total CDI	16	23	18	57
Admission	46,870	52,405	25,604	124,879
Patient days	281,187	286,699	143,580	711,466
Total CDI Rate per SMC admission (Total CDI Case per 10,000 admissions)	3.41	3.39	7.03	4.56
CO-CDI Rate per SMC admission (CO-CDI Case per 10,000 admissions)	0.21	1.34	3.91	1.44
CO-HCFA CDI Rate per SMC admission (CDI Case per 10,000 admissions)	0.43	0	0.39	0.24
HO-CDI Rate per SMC admission (HO-CDI Case per 10,000 admissions)	2.77	3.05	2.73	2.88
HO-CDI inpatient Incidence rate (HO-CDI Case per 10,000 patient days)*	0.46	0.56	0.49	0.5
Combined CDI inpatient incidence rate (HO-CDI + CO-HCFA, Case per 10,000 patient days)**	0.53	0.56	0.56	0.55

Patient characteristics

**Figure 2:**
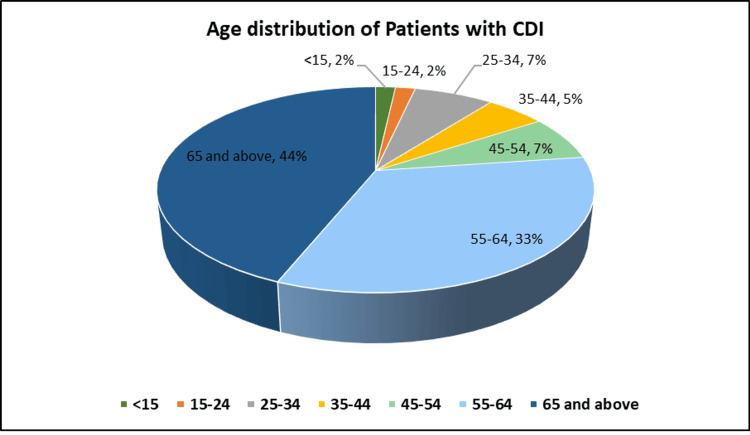
Age distribution of CDI patients in SMC, Bahrain SMC: Salmaniya Medical Complex; CDI: *Clostridioides difficile* infection

**Figure 3:**
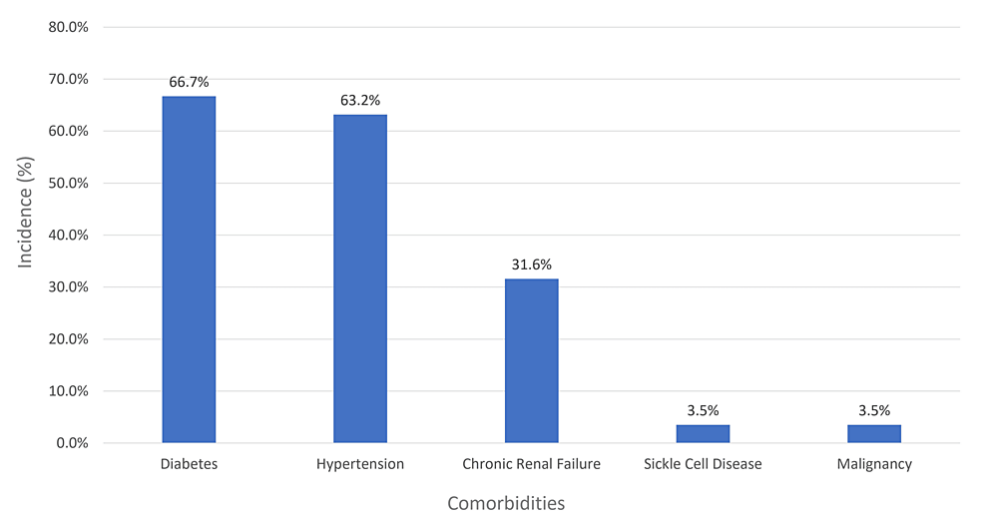
Comorbidities encountered among CDI patients in SMC, Bahrain SMC: Salmaniya Medical Complex; CDI: *Clostridioides difficile* infection

The median age of CDI patients was 61.8 years (standard deviation ± 17.8), with a balanced gender distribution (27 males, 47.4%; 30 females, 52.6%). Most diagnosed cases were on the medical floor (31 patients, 54.4%), followed by the surgical floor (nine patients, 15.8%), and the critical care unit (eight patients, 14.0%) (Figure 2). Diabetes and hypertension were the most common comorbidities, affecting 38 patients (66.7%) and 36 patients (63.2%), respectively (Figure 3).

CDI risk factors

At diagnosis, all 57 patients were undergoing acid-reducing therapy (proton pump inhibitors (PPIs)) and antimicrobial therapy (antibiotics or antifungals). Among them, three patients (5.2%) were receiving a combination of five different antimicrobial agents, six patients (10.5%) received four agents, 16 patients (28.1%) three agents, 23 patients (40.3%) two agents, and nine patients (15.8%) a single agent. Meropenem was the most frequently used antibiotic prior to CDI diagnosis (27 patients, 47.3%), followed by third-generation cephalosporins (13 patients, 22.8%) and penicillin (B-lactam/lactamase inhibitors) among six patients (12.3%).

Regarding CO cases, further analysis revealed that most (15 patients, 83.3%) had been exposed to antibiotics within three months before their CDI diagnosis. Second and third-generation cephalosporins were the most implicated antibiotics, used by 11 patients (73.3%), followed by penicillin (B-lactam/lactamase inhibitors) in five patients (33.3%).

Treatment and outcome of CDI

Treatment primarily consisted of metronidazole and oral vancomycin, administered in various formulations and regimens. Oral vancomycin alone was the most common treatment (26 patients, 46%), followed by a combination of intravenous (IV) metronidazole and oral vancomycin (14 patients, 25%) and oral metronidazole alone (eight patients, 14%). A combination of oral vancomycin and oral metronidazole was used in six patients (11%), while IV metronidazole alone was administered to three patients (5%).

Three patients (5.3%) developed acute toxic megacolon during their hospital stay but were managed conservatively. Another three patients (5.3%) experienced late CDI recurrence three months post-diagnosis. A majority (37 patients, 65%) were discharged alive, with an average hospital stay of 61 days post-CDI diagnosis. The mortality rate, defined as death during the hospital stay, was 35% (20 of 57 patients), with six deaths occurring within 30 days of CDI diagnosis and 14 thereafter.

**Figure 4:**
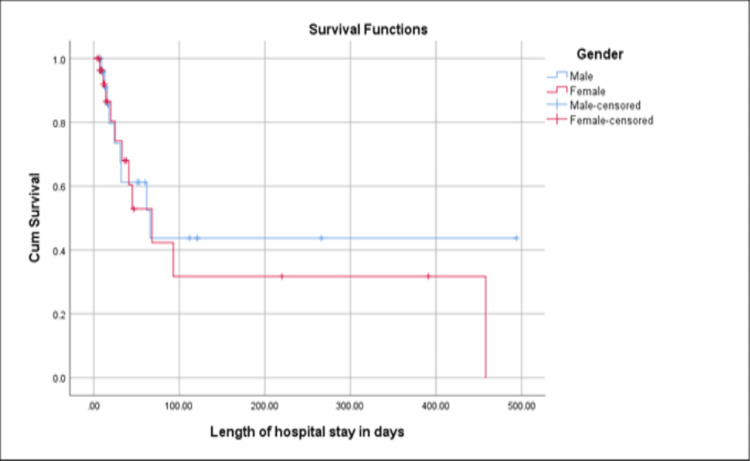
Survival plot between males and females in relation to the length of hospital stay Male mean survival time was 235.4 (std. error: 60.8) days Female mean survival time was 175.1 (std. error: 50.1) days Overall mean survival time was 207.1 (std. error: 41.9) days

**Figure 5:**
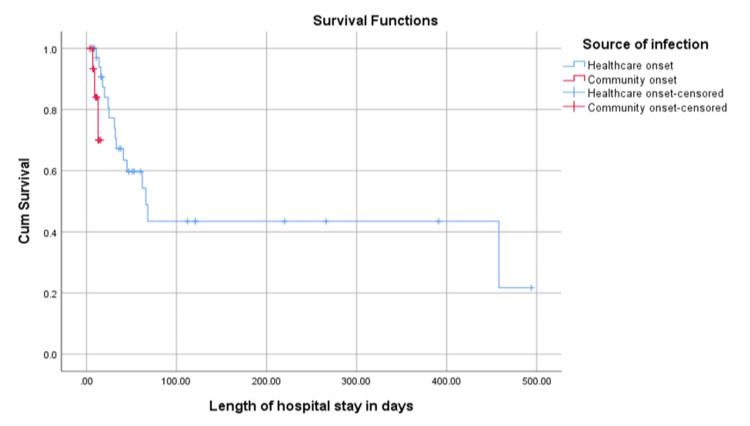
Survival plot between healthcare and community-onset in relation to length of hospital stay Healthcare onset mean survival time was 228.0 (std. error: 45.3) days Community onset mean survival time was 14.3 (std. error: 0.86) days Overall mean survival time was 215.2 (std. error: 43.2) days

Further analysis of the survival time with regard to gender and time of acquisition (HO Vs. CO) revealed that there was no statistical difference in survival time between the two genders based on Log-rank (Mantel-Cox) (p=0.584), but there was a significant difference in the survival time between CO and healthcare onset CDI patients where CO cases had a shorter survival time of 14.3 days, compared to 228.0 days among hospital-onset cases (p=0.025) (Figures 4, 5).

ATLAS score

**Figure 6:**
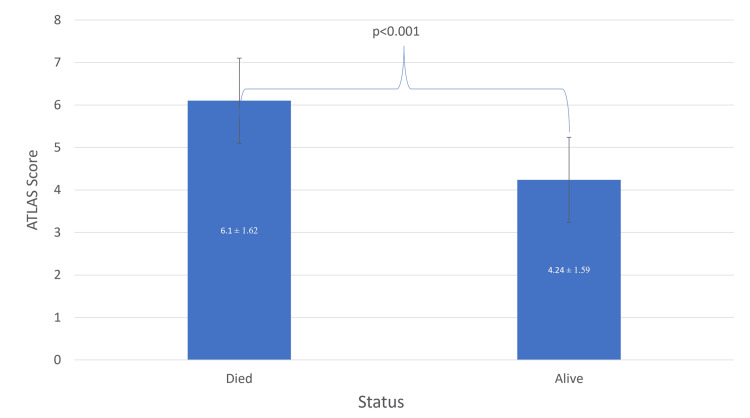
Association between ATLAS score and mortality rates in SMC, Bahrain ATLAS: Age, treatment with antibiotics, leukocyte count, albumin level, and serum creatinine; SMC: Salmaniya Medical Complex

The ATLAS scoring system was applied to evaluate patients with CDI (Table 2). All patients with an ATLAS score of two or less survived without complications or recurrence. Conversely, patients with an ATLAS score of nine all succumbed during their hospital stay. Figure 6 shows the outcomes distributed across different ATLAS score values. Univariate analysis suggested a possible correlation of mortality with age over 60, critical care admission, low albumin levels, and high ATLAS scores (Table 3). Multivariate regression analysis identified critical care admission and low albumin levels as independent risk factors for mortality (Table 4).

**Table 2:** ATLAS score and outcome of CDI in SMC, Bahrain ATLAS: Age, treatment with antibiotics, leukocyte count, albumin level, and serum creatinine; CDI: *Clostridioides difficile* infection; SMC: Salmaniya Medical Complex

ATLAS score	Patients in the defined score group, N (% total CDI cases)	Mortality N (%)	Toxic Megacolon N (%)	Recurrence N (%)
2	04 (7%)	0 (0%)	0 (0%)	0 (0%)
3	11 (19%)	1 (9%)	0 (0%)	0 (0%)
4	12 (21%)	2 (17%)	1 (8%)	0 (0%)
5	08 (14%)	4 (50%)	0 (0%)	0 (0%)
6	11 (19&)	6 (55%)	0 (0%)	1 (9%)
7	06 (11%)	3 (50%)	1 (17%)	2 (33%)
8	03 (5%)	2 (67%)	1 (33%)	0 (0%)
9	02 (4%)	2 (100%)	0 (0%)	0 (0%)
Total	57 (100%)	20 (100%)	3 (100%)	3 (100%)

**Table 3:** Relationship between mortality rates among the demographic and clinical characteristics of the patients diagnosed with CDI (N=57) † Some patients have multiple comorbidities § P-value has been calculated using Fischer’s Exact test ‡ P-value has been calculated using an independent sample t-test ** Significant at p<0.05 ATLAS: Age, treatment with antibiotics, leukocyte count, albumin level, and serum creatinine; CDI: *Clostridioides difficile* infection; SD: Standard deviation; WBC: White blood cell

Factor		Outcome		P-value §
		Died, n (%) (N=20)	Alive, n (%) (N=37)	
Age in years	≤60 years	03 (15.0%)	17 (45.9%)	0.023 **
	>60 years	17 (85.0%)	20 (54.1%)	
Gender	Male	09 (45.0%)	18 (48.6%)	1.000
	Female	11 (55.0%)	19 (51.4%)	
Location	Ward	08 (40.0%)	33 (89.2%)	<0.001 **
	Critical area	12 (60.0%)	04 (10.8%)	
Source of infection	Healthcare onset	16 (80.0%)	20 (54.1%)	0.120
	Community onset	03 (15.0%)	15 (40.5%)	
	Both	01 (05.0%)	02 (05.4%)	
Comorbidities †	Diabetes	15 (75.0%)	23 (62.2%)	0.389
	Hypertension	14 (70.0%)	22 (59.5%)	0.568
	Sickle cell disease	02 (10.0%)	0	0.119
	Malignancy	02 (10.0%)	0	0.119
	Chronic renal failure	08 (40.0%)	10 (27.0%)	0.377
Treatment	Vancomycin	11 (55.0%)	15 (40.5%)	0.622
	Metronidazole	03 (15.0%)	08 (21.6%)	
	Both	06 (30.0%)	14 (37.8%)	
Recent hospitalization	No	16 (80.0%)	33 (89.2%)	0.432
	Yes	04 (20.0%)	04 (10.8%)	
Length of hospital stay in days, mean ± SD		54.3 ± 97.8	62.0 ± 109.9	0.794
Laboratory Values	Serum albumin	24.8 ± 4.36	31.4 ± 5.36	<0.001 **
	Serum creatinine	179.5 ± 164.2	163.7 ± 201.9	0.766
	WBC count	15.7 ± 10.9	11.3 ± 7.24	0.077
	ATLAS score	6.10 ± 1.62	4.24 ± 1.59	<0.001 **

**Table 4:** Multivariate regression analysis to determine the significant independent risk factors associated with mortality among patients diagnosed with C. Diff (N=57) **Significant at p<0.05 AOR: Adjusted odds ratio; ATLAS: Age, treatment with antibiotics, leukocyte count, albumin level, and serum creatinine; CI: Confidence interval

Factor		AOR	95% CI	P-value
Age in years	≤60 years	Reference baseline	0.715-47.811	0.100
	>60 years	5.847		
Location	Ward	Reference baseline	4.459-824.7	0.002**
	Critical area	62.649		
Laboratory values	Serum albumin	1.362	1.048-1.771	0.021**
	ATLAS score	0.237	0.632-0.296	0.237

## Discussion

This study found that the incidence rate of healthcare facility-onset CDI (HO-CDI) at SMC is 0.5 per 10,000 patient days, notably lower than the international rates reported as 8.3 HO-CDI per 10,000 patient days [3,6,7]. This lower rate may reflect the effectiveness of our hospital’s infection control program, which includes an antibiotics stewardship program led by clinical pharmacists, immediate notification of all positive CDI cases by the microbiology laboratory to the infection control unit, daily rounds, and audits by the infection control team for positive CDI cases to ensure compliance with infection control measures. These measures include isolation precautions, periodic and terminal environmental cleaning with sporicidal agents for rooms of CDI patients, and adherence to hand hygiene practices by healthcare workers.

Although the number of HO-CDI cases remained stable throughout the study, This study observed an overall increase in CDI cases, mainly due to the rise in CO cases. Notably, a significant proportion of CO-CDI cases (83.3%) had been exposed to antibiotics within the three months preceding their CDI diagnosis, with cephalosporins being the most frequently implicated antibiotics. This finding aligns with previous studies that identified cephalosporin use during outpatient visits as a risk factor for CO-CDI [13].

Our findings showed no significant gender differences in CDI incidence, which was consistent with other research [14]. Additionally, 44% of our study population was over 65 years old (mean age: 61.8 years), supporting literature that identifies age as a significant risk factor for CDI development. Individuals over 65 years are at a 5-to-10-fold higher risk of contracting CDI compared to those under 65 [15-20].

All patients diagnosed with CDI in our study were on PPIs and antimicrobial therapies at the time of diagnosis. This supports existing literature indicating that hospitalized patients exposed to PPIs and antimicrobials are at an increased risk of developing CDI [21,22]. Previous studies have similarly identified antibiotic use, acid-reducing agent use, and inadequate hand hygiene as risk factors for CDI [23,24]. Barletta et al. (2013) and Alalawi et al. (2020) highlighted PPIs as having the highest risk for CDI, overshadowing age, sex, antibiotic use, and comorbidities, with no significant difference between PPI types [25,26]. Brown et al. (2014) discussed the link between the antibiotic used at CDI diagnosis and the duration of antibiotic use, noting an increased CDI risk with longer antibiotic duration [27].

Moreover, our study confirms the findings of Miller et al. (2013) [12], showing a significant correlation between higher ATLAS score values and mortality, with hypoalbuminemia being a primary risk factor for mortality among CDI cases. Critical care location was also associated with a higher mortality risk [11]. Further analysis of survival time revealed that CO cases had shorter survival times than hospital-onset cases. A potential explanation is that CO cases were more critical from the time of admission, but further studies might be needed to address all other factors contributing to shorter survival.

Limitations

Given its retrospective nature, this study may be subject to potential biases. Being a single-center study limits the generalizability of our findings to the broader population in Bahrain. However, our study lacked sufficient data on antibiotic duration to establish a correlation between antibiotic duration and CDI risk. Additionally, we lacked detailed information on drug exposure for CO-CDI cases, specifically outpatient PPI use before CDI development and the mode and duration of PPI administration for inpatients before CDI diagnosis.

## Conclusions

The incidence rate of HO-CDI in our center was 0.5 per 10,000 patient days, which is low compared to international rates; such a low rate is attributed to efficient infection control and antibiotic stewardship programs. However, having all CDI patients on antibiotics and acid suppression therapy at the time of diagnosis should direct our effort on enhancing awareness for healthcare professionals about CDI risk factors among hospitalized patients and advice about the need for periodic assessment of the necessity of such therapies with timely discontinuation when deemed unnecessary. The overall CDI rate increased during the study period, driven by a rise in CO cases; further investigating the risk factors among this category in our study revealed that most patients were exposed to antibiotic therapy within the past three months of their CDI diagnosis.

In agreement with other studies, the ATLAS score was found to be a useful tool for routine evaluation of patients with CDI. Admission in critical care and hypoalbuminemia were the most important poor prognostic factors among our inpatient participants with positive CDI, as both were significantly associated with higher mortality.

Future studies should aim to identify risk factors for CDI acquisition in the community, concentrating on acid suppressant exposure and further analysis of outpatient antibiotics use with revelation of antibiotics prescribing behaviors among health care professionals and restriction of dispensing antibiotics from outpatient pharmacies. Additionally, conducting further multi-center studies in Bahrain will be of great value in establishing the national incidence and prevalence rate of CDI at both community and healthcare facility levels.
